# Powering precision: development of a certified reference material for elemental composition analysis of lithium nickel manganese cobalt oxide (Li-NMC) cathode material for lithium-ion batteries

**DOI:** 10.1007/s00216-025-05766-7

**Published:** 2025-02-10

**Authors:** Sebastian Recknagel, Silke Richter, Marion Hoppe, Angela Meckelburg, Carsten Prinz, Janina Roik, Carlos Abad

**Affiliations:** Bundesanstalt für Materialforschung und -prüfung (BAM), Richard-Willstätter-Str. 11, 12489 Berlin, Germany

**Keywords:** Certified reference material, Lithium nickel manganese cobalt oxide (Li-NMC), Lithium-ion battery, Cathode material

## Abstract

This work presents the development and certification of the world’s first certified reference material (CRM), BAM-S014, for a lithium nickel manganese cobalt oxide (LiNi_0.33_Mn_0.33_Co_0.33_O_2_ or Li-NMC 111) cathode material—an integral component in high-energy-density lithium-ion batteries that power electric vehicles (EVs), portable electronics, and stationary energy storage systems. By providing certified mass fractions for 11 elements, this CRM addresses a critical need for accurate and traceable elemental analysis, supporting quality control and regulatory compliance in the global battery industry. Ensuring reliable and harmonized measurements supports the efficient use of resources, including the reuse of recycled materials, and ultimately helps maintain product performance and safety. The values were assigned through an interlaboratory comparison involving 16 participating laboratories and various analytical techniques such as inductively coupled plasma optical emission spectrometry (ICP-OES), inductively coupled plasma mass spectrometry (ICP-MS), X-ray fluorescence (XRF) spectrometry, and combustion analysis. In addition to chemical characterization, the certification process includes homogeneity and stability testing of the candidate material. Details are provided on the analytical methods used for chemical characterization and the calculation of the uncertainties of the certified mass fractions. In addition to detailing the development of the CRMs, this work provides an overview of ongoing standardization activities in Li-component analysis, thereby guiding the calibration of analytical methods and contributing to the establishment of globally accepted standards for evaluating energy storage materials and advancing sustainable mobility and clean energy solutions.

**Graphical Abstract:**

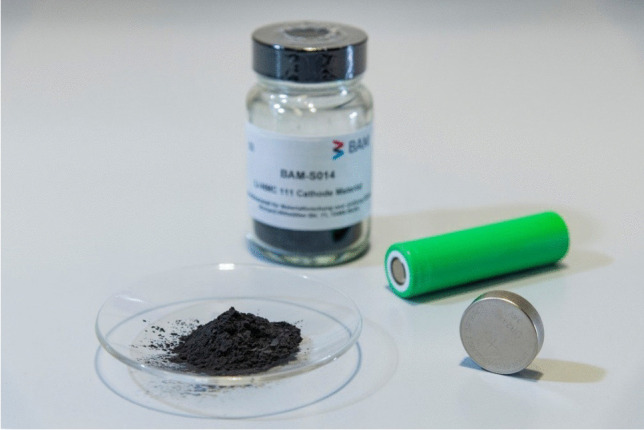

## Introduction

Lithium-ion batteries have emerged as the leading technology for portable electronic devices, electric vehicles, and energy storage systems for power grids due to their high energy density, long cycle life, and low self-discharge rate [1, 2] and lightweight design. The cathode material plays a crucial role in the performance and safety of lithium-ion batteries.

Among the various cathode materials employed in lithium-ion batteries, Li-NMC compositions have gained prominence due to their balanced performance characteristics. Structurally, NMC cathode materials are layered oxide compounds formed from nickel (Ni), manganese (Mn), and cobalt (Co), where each element contributes to the overall electrochemical performance and stability of the material. Compared to lithium iron phosphate (LFP)–based cathodes, Li-NMC materials generally offer higher energy densities, resulting in longer ranges for electric vehicles and longer runtimes for portable electronics [1, 3]. While LFP cathodes offer advantages such as greater thermal stability and material abundance, Li-NMC cathodes are favored in applications where energy density and performance are critical. The numerical suffix after the NMC labelling (e.g., NMC 111, NMC 532) indicates the proportion of these elements in the cathode. The combination of nickel, manganese, and cobalt in Li-NMC provides improved energy density and rate capability, making it a versatile choice that can be customized by adjusting the relative proportions of these transition metals.

From a resource availability perspective, Li-NMC cathodes rely on elements such as cobalt, nickel, and manganese, whose supply chains face certain challenges due to geopolitical factors and variable ore grades. However, ongoing efforts to diversify sourcing, improve extraction technologies, and enhance global trade policies are helping stabilize these supply chains [4]. In parallel, recent trends in reducing cobalt content (e.g., from NMC 111 to NMC 622 or 811) aim to minimize the reliance on this relatively rare and ethically challenging resource [5, 6]. This strategy mitigates supply risk, lowers material costs, and potentially reduces the environmental footprint of mining and refining cobalt.

Furthermore, Li-NMC materials can be recovered and recycled from end-of-life batteries. Advanced recycling technologies are emerging to efficiently reclaim lithium, nickel, manganese, and cobalt for reintegration into new battery production streams [7]. This circular approach not only reduces dependence on raw material extraction but also contributes to a more sustainable lifecycle for lithium-ion batteries, ultimately supporting the development of a robust and environmentally responsible energy storage infrastructure.

Elemental analysis of cathode materials is critical to ensuring consistent quality and optimal performance of lithium-ion batteries, especially when recycling materials are used to produce new batteries [8]. It helps to ensure the consistency and quality of the material, which ultimately impacts the performance and safety of the battery. The elemental composition of the cathode material must be accurately determined to optimize the battery’s performance, meet the regulatory requirements [9, 10], and ensure consistency across manufacturing facilities [11].

In addition to elemental analysis, other material properties — such as lithium isotopic distribution, particle size, and internal surface area — can also significantly impact battery performance and safety. Lithium isotopes provide a unique method for tracing the provenance of lithium, which is essential for both resource sustainability and performance optimization [12]. This is particularly relevant as digital battery passports (DBPs) are becoming increasingly important tools for tracking the lifecycle and provenance of raw materials, including lithium [13].

A certified reference material (CRM) is critical in this context as it provides a standard against which elemental composition can be measured to ensure that batteries consistently meet safety and performance criteria across laboratories. Without such a reference material, variations in analytical methods between laboratories could lead to discrepancies that could affect the quality and safety of the final product. The use of CRM enables reproducibility, comparability, and confidence in the data across different industries and research areas, supporting the continuous development of safe and efficient battery technologies.

Several methods are available for elemental analysis of metals in cathode materials for lithium-ion batteries. The most common methods include ICP-OES, ICP-MS, XRF, and AAS. Due to the high technical importance of Li-based battery components, there are currently several standardization activities regarding analysis methods for characterizing them. The technical committee of the International Organization for Standardization ISO TC 333 (lithium) is dealing with a couple of projects related to analytical methods on raw materials (Li-chloride, Li-carbonate) and battery materials (see Table 1). The ISO 12467 series is particularly relevant to the analysis of lithium-based cathode materials.

**Table 1:** Standardization projects for analytical methods within ISO TC 333

ISO/CD 10655	Methods for analysis of lithium hexafluorophosphate — Determination of metal ions content by inductively coupled plasma optical emission spectrometry (ICP-OES)
ISO/WD 10662	Determination of main content of lithium carbonate-Potentiometric titration
ISO/AWI 11045–1	Methods for chemical analysis of lithium salts — Part 1: Quantitative determination of lithium hydroxide and lithium carbonate content in lithium hydroxide monohydrate — Potentiometric titration method
ISO/WD 11757	Lithium carbonate — Determination of elemental impurities by ICP-OES
ISO/WD 12380	Lithium carbonate — Determination of insoluble particles in acid by gravimetry
ISO/WD 12386	Lithium carbonate — Determination of metallic magnetic impurities by ICP-OES
ISO/WD 12403	Lithium carbonate — Determination of chloride content by potentiometry
ISO/CD 12467–1	Chemical analysis of lithium composite oxides — Part 1: Determination of main components
ISO/AWI 12467–2	Chemical analysis of lithium composite oxides — Part 2: Determination of trace elements
ISO/AWI 12467–3	Chemical analysis of lithium composite oxides — Part 3: Determination of lithium carbonate and lithium hydroxide contents
ISO/WD 16398	Lithium chloride — Determination of impurities — ICP-OES method
ISO/AWI 16423	Lithium hydroxide monohydrate — Determination of impurities — ICP-OES method
ISO/AWI 24991	Methods for chemical analysis of lithium concentrates — Determination of lithium oxide content — Flame atomic absorption spectrometry
ISO/AWI 24992	Methods for analysis of lithium hexafluorophosphate — Determination of anions content by ion chromatography (IC)
ISO/PWI 20307	Lithium carbonate — Determination of lithium content — ICP-OES method using an internal standard element

CRMs are essential for accurate and reliable elemental analysis of cathode materials. They are used to calibrate and validate analytical methods, ensure the accuracy of results, and compare the performance of different laboratories. The lack of reference materials in the field of Li-based cathode materials was the reason to produce the NMC CRM BAM-S014. The international interlaboratory test database EPTIS (www.eptis.bam.de) does not list any interlaboratory tests in the field of chemical characterization of Li-based cathode materials. Apart from BAM-S014, no other CRMs were available for the international interlaboratory validation of the draft standard ISO 12467. Only one candidate reference material NMC-811 was analyzed in addition to BAM-S014. In the field of raw materials for the production of Li batteries, projects for the certification of lithium carbonate reference materials are underway at BAM.

Preparing and certifying CRMs for elemental analysis of cathode materials require a rigorous and well-defined process. The development of CRMs also supports global standardization efforts, promoting uniformity in battery performance standards internationally, which is critical given the global nature of the battery market [14].

The standards ISO 17034 and ISO 33405 provide guidance for the production of CRMs [15, 16]. These standards specify the requirements for the homogeneity and stability testing and characterization/value assignment of CRMs. They also provide guidelines for the design and execution of round-robin tests to evaluate the performance of laboratories and validate the CRM's properties.

In this work, we developed a CRM for the elemental composition of Li-NMC cathode material, BAM-S014. The material was characterized for its elemental composition, the isotopic ratio of Li, the specific surface area, and the particle size distribution. The certification was carried out in a round-robin test with external laboratories to evaluate the composition and additional properties of the material.

The results of the round-robin test demonstrated that the CRM is suitable for the accurate and reliable determination of elemental composition in Li-NMC cathode materials. The CRM showed excellent homogeneity, and the analytical results obtained from different laboratories were consistent and comparable.

## Materials and methods

### Material preparation

A total of 25 kg of NMC 111 powder, produced by and obtained from MEET—Münster Electrochemical Energy Technology (University of Münster, Germany), was taken as candidate material.

The process of synthesizing battery cathode materials using coprecipitation involves two main steps: coprecipitation of transition metals and sintering. The first step involves mixing transition metals in the required proportion to form an ionic solution and coprecipitating these metals under a controlled environment normally by adjusting pH to obtain uniform particles, which are also known as precursors. A chelating agent is normally used during the precipitation process to obtain single-phase coprecipitates. The obtained precursors are washed with deionized (DI) water to remove impurities and dried at room temperature or higher to remove residual water and/or other solvents that are used to clean the coprecipitates. The coprecipitate was ground before mixing with a lithium source such as lithium hydroxide (LiOH) or lithium carbonate (Li_2_CO_3_). The homogeneous mixture of coprecipitate and lithium source is sintered in one or two steps depending on the metal sources and sintering conditions to obtain the active materials for battery electrodes [17]. The material was carefully mixed in BAM to ensure its homogeneity. A total of approximately 1000 units of 25 g each were filled into amber glass bottles under argon and sealed with an air-tight aluminum sealing lid below the screw cap.

### Homogeneity of the material

Of the total batch of 25 kg of candidate material, which ultimately resulted in 1000 units of the reference material, 18 units of 25 g NMC 111 each were tested for homogeneity. This corresponds to 1.8% of the total batch. In contrast, ISO 33405 requires 10 units as the minimum quantity for homogeneity testing. From each of the 18 bottles, two sub-samples were taken for homogeneity measurements. The main components, Co, Ni, and Mn, were determined using a wavelength dispersive X-ray fluorescence spectrometer (MagiX Pro, Panalytical, Almelo, Netherlands) equipped with a rhodium tube (tube voltage for Co, Ni: 60 kV, for Mn: 57 kV) and a LiF200 crystal. All elements were measured using their K_α_-lines with a measuring time of 30 s and a background measuring time of 5 s. All XRF-measurements were carried out on pressed powder pellets. The test samples were pressed in Al-cups on a 200-kN press to pellets with a thickness of 5 mm and a diameter of 32 mm (pressing time 10 s). One of the pellets was randomly chosen as drift control sample. This sample was measured 10 times before and five times during the whole measurement sequence.

Homogeneity measurements for the elements Li, Al, Cr, Fe, Na, S, and Si were carried out using ICP-OES (Spectro Arcos, Kleve, Germany) following the procedure described in ISO/CD 12467–1 and 12467–2 [18, 19]. For this purpose, 10 mg (Li determination) or 1000 mg (determination of trace elements) of NMC 111 were weighed and mixed with 10 mL of aqua regia (HCl, p.a. and HNO_3_, p.a., Merck KGaA, Darmstadt, Germany) and heated on the hotplate (IKA-Werke, Staufen, Germany) at 120–150 °C. After complete dissolution and cooling, the solutions were filled up to 100 mL in quartz flasks and measured against standard solutions (matrix matched with Li, Co, Ni, and Mn for the trace elements).

Homogeneity measurements for carbon were performed using a C/S-analyzer Elementrac CS-I (Eltra, Haan, Germany). Each 700 mg sample material was weighed into a pre-annealed ceramic crucible, mixed with 0.7 g Fe and 1.5 g W (Eltra, Haan, Germany) and burnt in an induction furnace under an oxygen atmosphere. The resulting CO_2_ was detected using the integrated IR detector.

### Stability of the material

**Fig. 1:**
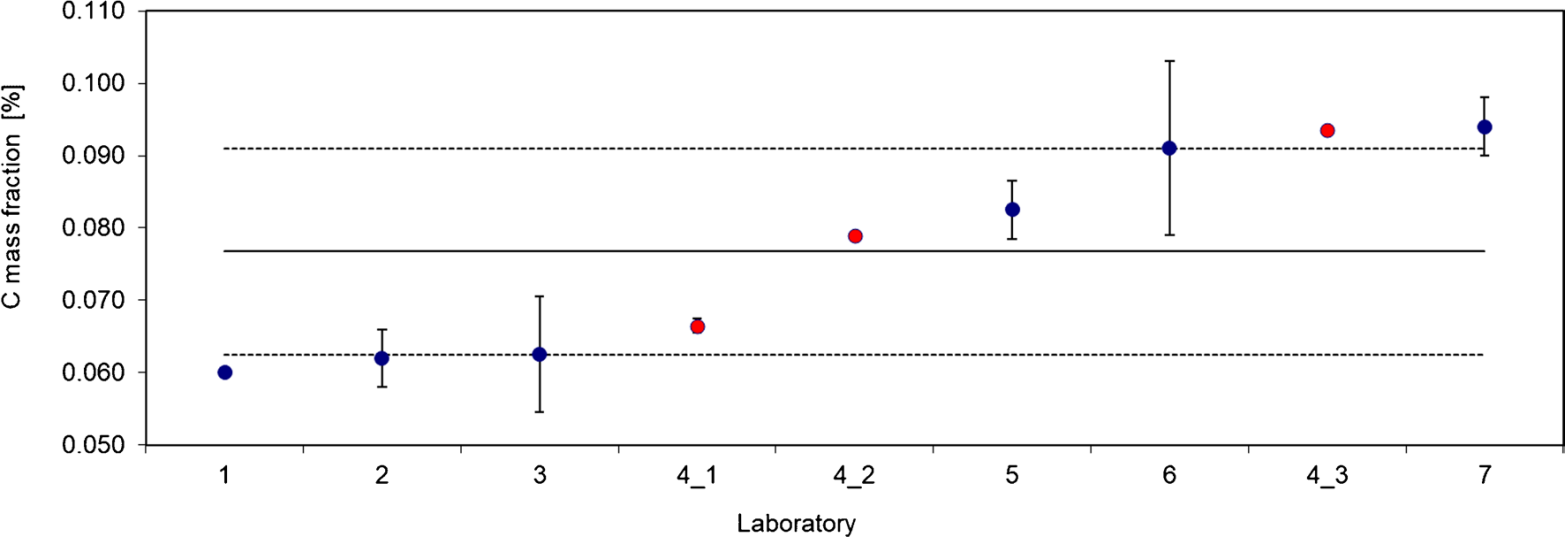
Carbon content in NMC 532, preceding interlaboratory comparison. Laboratory 4 determined the C content three times within 5 months (4_1, 4_2 (3 months), 4_3 (5 months))

Due to its chemical composition, Li-NMC should be stable, which is an important prerequisite for use in lithium batteries. However, Shizuka et al. describe the uptake of CO_2_ by NMC-cathode materials [20]. In a study on the analysis of NMC 532 that preceded the certification project, an increase in the carbon content over time was also observed (Fig. 1).

Within 5 months, the carbon content determined by Laboratory 4 increased from 0.066 to 0.094% mass fraction. No special storage conditions were implemented to prevent air access to the sample material during this period. As the oxygen content is 33%, an increase in this element due to CO_2_ uptake could not be observed. A similar increase of carbon content could be observed in the candidate material for BAM-S014 (NMC 111). One of the participating laboratories measured carbon twice every 7 days and found 0.056% on the first day and 0.062% 7 days later when the sample was stored in air.

Therefore, the candidate material was stored under argon atmosphere and the sample bottles were filled under argon. The average carbon content determined in the interlaboratory comparison is stated in the certificate as an informative value only.

In the case that the material absorbs CO_2_, the total mass of the sample and therefore the content of the elements it contains will also change slightly. To take this into account, a worst-case estimate that the content of C doubles from 0.06 to 0.12% was assumed. The effects on the certified contents of the other elements were calculated and considered in the respective uncertainties of the certified values. In the certificate of the CRM, it is stated that access of air to the material must be avoided.

### Characterization and value assignment

#### Determination of elemental composition

A total of 16 laboratories from industry and research participated in the interlaboratory comparison for certification. The participating laboratories took part in a subsequent interlaboratory comparison on a Li-NMC material to qualify for the certification project. The laboratories were asked to take six test portions for analysis. They were free to choose any suitable method for analysis. Almost all participants used a mixture of HCl and HNO_3_ to dissolve the sample. Some of them added HF to the acid mixture. This procedure had already been extensively tested on NMC materials in the previous qualification round-robin test and in preliminary work for the draft ISO 12467 standards [18, 19] and was found to be suitable. The final determination was carried out in almost all cases using ICP-OES or ICP-MS. Two laboratories used XRF of borate beads, one to determine Ni, Co, and Mn and the other for Na. Significant differences between the different analysis methods were not observed. To ensure traceability of the analytical results, laboratories were instructed to use only calibrants prepared from pure metals or stoichiometric compounds or traceable commercial calibration solutions. Table 2 gives an overview of the methods and procedures used for the determination of metallic main components and traces including sulfur. Calibration was mostly performed using commercial mono-element calibration solutions from different manufacturers. Some of the laboratories used matrix-matched standard solutions (Li, Mn, Co, Ni) when determining the traces. Internal standardization with Sc or In was used by several participants. The sample intakes for the main components Li, Mn, Co, and Ni were between 20 and 500 mg and for the trace elements between 50 and 1000 mg.

**Table 2:** Analytical methods used to analyze BAM-S014 (metallic compounds, sulfur)

Laboratory	Elements	Sample intake (g)	Sample pretreatment	Analytical method/calibrant
NMC-01	Li, Ni, Mn, Co	0.5 g	Dissolution in HCl/H_2_O_2_	ICP-OES/standard solutions (Merck KGaA, Darmstadt, Germany)
NMC-01	Al, Cr, Cu, Fe, Na, P, S, Si, Ti, V	0.5 g	Dissolution in HCl/H_2_O_2_	ICP-MS/standard solutions (LabKings B.V., Hilversum, The Netherlands)
NMC-02	Li, Ni, Mn, Co	0.02 g	Dissolution in aqua regia (ISO/CD 12467–1 [18])	ICP-OES
NMC-02	Al, Cr, Cu, Fe, Na, P, S, Si	1 g	Dissolution in aqua regia (ISO/AWI 12467–2 [19])	ICP-OES/ICP-MS (2 datasets for some elements)/standard solutions (Merck KGaA, Darmstadt, Germany)
NMC-03	Li, Ni, Mn, Co	0.05 g	Dissolution in aqua regia	ICP-OES/standard solutions (VWR International, LLC, Radnor, USA)
NMC-03	Al, Cu, Fe, Na, S, Si	0.1 g	Dissolution in aqua regia	ICP-OES
NMC-05	Li, Ni, Mn, Co	0.115 g	Dissolution in aqua regia	ICP-OES/standard solutions (Merck KGaA, Darmstadt, Germany)
NMC-05	Al, Fe, Na, S, Si	1 g	Dissolution in aqua regia	ICP-OES/standard solutions (Merck KGaA, Darmstadt, Germany)
NMC-07	Li, Ni, Mn, Co, Cu, Na, Si	0.5 g	Microwave pressure digestion	ICP-OES/pure substances
NMC-08	Li, Ni, Mn, Co	0.05 g	Dissolution in HCl/HNO_3_	ICP-OES/standard solutions traceable to NIST-SRMs
NMC-08	Al, Cu, Fe, Na	0.05 g	Dissolution in HCl/HNO_3_/HF	ICP-OES/standard solutions traceable to NIST-SRMs
NMC-09	Li, Ni, Mn, Co	0.1–0.4 g	Dissolution in 6 mL HCl/2 mL HNO_3_ at 80 °C (4 h)	ICP-OES/standard solutions (CPAchem Ltd, Bogomilovo, Bulgaria)
NMC-09	Al, Cr, Cu, Fe, Na, P, S, Ti	0.1–0.4 g	Dissolution in 6 mL HCl/2 mL HNO_3_ at 80 °C (4 h)	ICP-OES/standard solutions (CPAchem Ltd, Bogomilovo, Bulgaria)
NMC-09	Li	0.1–0.4 g	Dissolution in 6 mL HCl/2 mL HNO_3_ at 80 °C (4 h)	ICP-MS/standard solutions (CPAchem Ltd, Bogomilovo, Bulgaria)
NMC-09	Ni, Mn, Co	0.05 g	Fusion with Li-tetraborate, Li-metaborate, LiBr	XRF/pure substances
NMC-10a	Li, Ni, Mn, Co	0.05 g	Microwave pressure digestion	ICP-OES
NMC-10b	Li	0.05 g	Microwave pressure digestion	ICP-OES
NMC-11	Li, Ni, Mn, Cu, Na	0.1 g	Dissolution in acid	ICP-OES
NMC-12	Li, Ni, Mn, Co	0.125 g	Dissolution in aqua regia	ICP-OES/standard solutions (Bernd Kraft, AnalytiChem GmbH, Duisburg, Germany)
NMC-12	Al, Cr, Cu, Si, V	0.25 g	Dissolution in aqua regia	ICP-OES/standard solutions (Bernd Kraft, AnalytiChem GmbH, Duisburg, Germany)
NMC-13	Li, Ni, Mn, Co	0.05–0.1 g	Acid pressure digestion with HCl/HNO_3_	ICP-OES
NMC-14	Li, Ni, Mn, Co	0.1 g	Dissolution in aqua regia at 90 °C (2 h)	ICP-OES/standard solutions (Merck KGaA, Darmstadt, Germany)
NMC-15	Na	0.5 g	Li_2_B_4_O_7_ glass bead	XRF

Table 3 gives an overview of the methods and procedures used for the determination of non-metallic constituents O, N, and S. The participating laboratories used combustion instruments from different manufacturers for C and S determination and carrier gas hot extraction instruments for the determination of O. The sample intakes were between 0.2 and 150 mg for oxygen and between 10 and 300 mg for carbon and sulfur. Calibration was performed using pure substances such as oxides, carbonates and sulfates, or steel CRMs from different manufacturers. As for the metals, no dependence of the internal laboratory scatter on the sample mass used for the determination of the non-metallic contents could be observed.

**Table 3:** Analytical methods used to analyze BAM-S014 (non-metals)

Laboratory	Elements	Sample intake (g)	Additives	Analytical method
NMC-01	O	0.15 g	Ni-capsule	Carrier gas hot extraction, calibration with SiO_2_
NMC-01	C, S	0.25 g		Combustion/IR, calibration with steel standards^a^
NMC-02	O	0.2 mg	Sn-capsule	Carrier gas hot extraction, calibration with Fe_2_O_3_
NMC-02	C, S	0.1 g	2 g W + 1 g Fe	Combustion/IR, calibration with BaCO_3_ (carbon) and BaSO_4_ (sulfur)
NMC-04	O	5 mg	Sn-capsule + graphite	Carrier gas hot extraction, calibration with steel CRM (JK47)
NMC-04	C, S	0.01 g	1.5 g W/Sn	Combustion/IR, calibration with steel CRM (ECRM 284–2)
NMC-05	C	0.01 g	W/Sn + Fe	Combustion/IR, calibration with steel standards^b^
NMC-06	O	3.5 mg	Ni-capsule, Sn	Carrier gas hot extraction, calibration with ZrO_2_
NMC-06	C, S	0.2 g	Fe and W/Sn	Combustion/IR, calibration with steel-CRM (ECRM 031–3)
NMC-07	O	0.15 g		Carrier gas hot extraction
NMC-07	C, S	0.3 g		Combustion/IR, calibration with pure substances
NMC-08	O	8 mg	Ni/Sn	Carrier gas hot extraction, calibration with ZrO_2_
NMC-08	C, S	0.05 g	W18/Sn5	Combustion/IR, calibration with CO_2_ (carbon) and K_2_SO_4_ (sulfur)
NMC-09	O	2 mg	Ni-capsule, Sn, graphite	Carrier gas hot extraction, calibration with TiO_2_, ZrO_2_
NMC-09	C, S	0.15 g	Additives: W/Sn	Combustion/IR, calibration with steel CRM (BAM)
NMC-11	O	5–10 mg		Carrier gas hot extraction, calibration with ZrO_2_
NMC-11	C, S	0.3 g		Combustion/IR, calibration with steel CRM^b^
NMC-15	S	0.2 g		Carrier gas hot extraction, calibration with steel CRM^c^
NMC-17	O	60 mg		Carrier gas hot extraction, calibration with ZrO_2_ and WO_3_
NMC-17	C, S	0.15 g		Combustion/IR, calibration with steel CRM^b^

^a^Steel CRM (Brammer Standard Comp. Inc., Houston, USA)

^b^Steel CRM (Leco Corp., St. Joseph, USA)

^c^Steel CRM (Eltra, Haan, Germany)

#### Determination of lithium isotope ratio

The lithium isotope ratio (^7^Li/^6^Li) was determined by one laboratory. The mean value is 12.34 with a standard deviation of 0.01. Three of the datasets were obtained by measuring the samples against isotopic standards (NIST SRM 8545 and JRC IRMM-016) using a continuum source atomic absorption spectrometer model contrAA800D HR-CS-AAS (Analytik Jena, Germany) with GF (PIN platform) as described in [21] using different sample pretreatment procedures with and without ion exchange matrix separation. The fourth dataset was obtained by multicollector inductively coupled plasma mass spectrometry (MC-ICP-MS, Thermo Neptune Plus, Thermo Fisher Scientific, Bremen, Germany) measurements against isotopic standards [21].

#### Determination of specific surface area A_BET_

One of the participants determined the specific surface area of BAM-S014 with an automated surface area and porosity analyzer ASAP 2020 (Micromeritics, Norcross USA). Krypton was used as adsorption gas at 77 K to measure the adsorption branch up to p/p_0_ 0.5. Other gases than krypton, e.g., nitrogen, are not suitable for the determination of a specific surface area of 0.5 m^2^/g. Therefore, the results of two other laboratories using nitrogen were removed.

Prior to measurement, sample preparation was performed including outgassing in vacuum with a final pressure of below 10 Pa. The sample was then heated in vacuum at a rate of 5 K/min to 473.15 K and held at 473.15 K for at least 5 h. Afterwards, the sample was cooled slowly to ambient temperature.

The supported value of specific surface area is calculated with the multipoint data analysis method according to Brunauer, Emmet, and Teller (BET) [22] and according to ISO 9277 [23] by using the linear form with a minimum of five adsorption points in a relative pressure range of 0.03 ≤ p/p_0_ < 0.14. The value used for the cross-sectional area of krypton was 0.202 nm^2^, and the sample intake was 3.0 to 3.5 g.

#### Determination of particle size

In addition to the chemical composition, seven of the participating laboratories determined the particle size distribution of the material. 0.4–1 g of powder was dispersed in a specific liquid (water, Na_4_P_2_O_7_-solution, Tris(nonylphenyl)phosphite) using ultrasound. To detect the particle size, all laboratories used laser diffraction according to ISO 13320 [24] using the Fraunhofer approximation for calculation. Instruments used were Mastersizer 3000 (Malvern Panalytical, Almelo, The Netherlands), Beckman Coulter LS 13320 (Beckman Coulter, Inc., Brea, USA), and HELOS/KF-Magic (Sympatec GmbH, Clausthal-Zellerfeld, Germany).

## Results and discussion

The certification campaign of BAM-S014 included homogeneity assessment, stability testing, an interlaboratory comparison with external laboratories for characterization to assign certified values, and calculation of the total uncertainty budget also enabling a statement of traceability.

### Assessment of homogeneity

The inhomogeneity contributions *u*_bb_ potentially hidden by the measurement uncertainty and to be included into the total uncertainty budget of the certified properties were estimated according to ISO Guide 35 (now ISO 33405 [16]) as the maximum of the values obtained from Eqs. (1) and (2).

1 $${s}_{\text{bb}}=\sqrt{\frac{MS{\text{among}}\hspace{0.33em}\hspace{0.33em}-MS{\text{within}}}{n}}$$

2 $${u}_{\text{bb}}^{*}=\sqrt{\frac{MS{\text{within}}}{n}}\hspace{0.33em}\sqrt[4]{\frac{2}{N(n-1)}}$$

where

*MS*_among_mean of squared deviations between bottles (from 1-way ANOVA)

*MS*_within_mean of squared deviations within bottles (from 1-way ANOVA)

*n*number of replicate sub-samples per bottle

*N*number of bottles selected for homogeneity study

*s*_bb_ signifies the between-bottle standard deviation, whereas ${u}_{bb}^{*}$ denotes the maximum heterogeneity that can potentially be hidden by an insufficient repeatability of the applied measurement method (which must be considered as the minimum uncertainty contribution). In any case, the larger of the two values was used as *u*_bb_. Equation (1) does not apply if *MS*_within_ is larger than *MS*_among_, which was the case for Cr and Na.

The only element for which potential inhomogeneity accounts for the greatest uncertainty is lithium. However, the sample weight used for the homogeneity analysis for lithium was significantly less than for all other elements (only about 1%). The main component Li could not be analysed by XRF but by ICP-OES; this and the small sample weight lead to a larger scatter in the homogeneity analysis and thus simulate a larger inhomogeneity.

### Elemental composition

Sixteen laboratories reported results for element determination in the candidate material. All data were thoroughly evaluated for technical outliers and subsequently tested for statistical outliers (Grubbs, Dixon, Cochran) using the software program eCerto [26]. The original dataset of each parameter is tested for outlying and straggling results representing levels of significance of 1% and 5%, respectively. One outlying value for Ni (Grubbs 95%) and two for Na (Grubbs Pair 99%) were detected. These outlying results were removed from the dataset after discussion in a board meeting with the participating laboratories. One laboratory withdrew all their results because of technical problems during the analysis. The datasets accepted for the calculation of the certified mass fractions are shown in Table 4; Table 5 shows the data provided for information only. There may be several reasons for providing data for information purposes only. In the case of carbon, the potential increase of the content due to CO_2_ absorption is the reason for not certifying its mass fraction. For phosphorus, titanium, and vanadium, the number of datasets was too small and for silicon the range of results was too large.

**Table 4:**
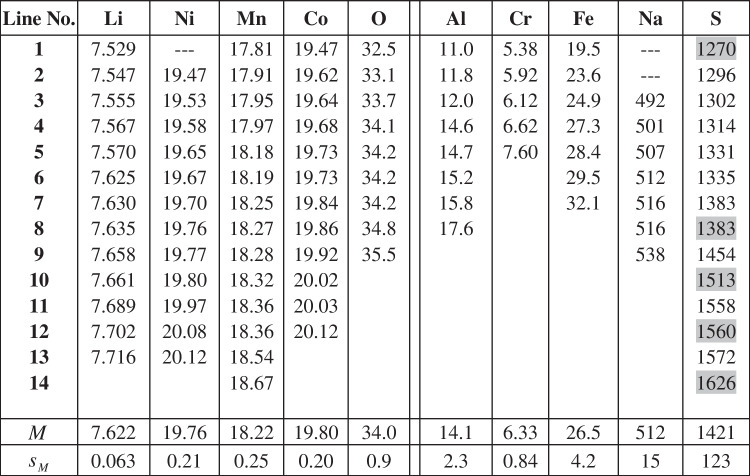
Results of elemental analyses (mass fractions in % (Li, Ni, Mn, Co, O) or mg/kg (Al, Cr, Fe, NA, S)), certified values (gray, ICP-OES results for S)

The standard deviation of the laboratory averages for the main constituents Li, Ni, Mn, and Co is 1% relative. In particular, the two elements Ni and Co, which determine the value in subsequent recycling, are analyzed with sufficient precision. The precision obtained also reflects the state of the art in laboratory interlaboratory comparisons. For the minor and trace elements, the standard deviations of the laboratory mean values are between 3% (Na) and 16% (Al, Fe), which is also acceptable in view of their content. Sulfur was analyzed by two fundamentally different methods: wet chemical with ICP-OES after digestion and combustion analysis without prior digestion. Both methods gave consistent results, and no method-dependent influence could be detected.

**Table 5:** Results of elemental analyses (mass fractions in mg/kg), informative values

Line no	C	P	Si	Ti	V
**1**	501	10.9	9.0	0.622	0.04
**2**	520	11.4	18.1	0.717	1.3
**3**	584	14.4	64.3		
**4**	585		65.2		
**5**	589		76.5		
**6**	603		88.5		
**7**	616				
**8**	672				
**9**	729				
$M$	600	12.2			
${s}_{M}$	70	1.9			

### Specific surface area A_BET_

Three laboratories reported specific surface area results but only one of them used krypton as the adsorption gas which is necessary for small surface areas. Therefore, only the results from this laboratory are used to calculate the information value given on the BAM-S014 certificate. Table 6 shows the individual results of the BET measurements.

**Table 6:** Results of specific surface measurements

No	Specific surface area, m^2^/g	*c*-value	Correlation coef	p/p_0_ range
1	0.4974	115.99	0.999933	0.0325–0.1347
2	0.4920	105.65	0.999920	0.0326–0.1353
3	0.4792	98.74	0.999933	0.0326–0.1347
4	0.4987	110.21	0.999926	0.0326–0.1350
5	0.4584	92.84	0.999924	0.0325–0.1347
6	0.4918	116.31	0.999928	0.0326–0.1357
7	0.4899	114.67	0.999939	0.0326–0.1346
Mean value	0.487			
Standard dev	0.014			
2* s*	0.028			

### Particle size

Table 7 shows the results obtained by the various laboratories determining the particle size distribution of the material. Compared to the results of the certification interlaboratory comparison of BAM-D001, a SiC reference material with certified values for the parameters d_10_, d_50_, d_90_ the results for BAM-S014 are quite good. The relative standard deviations for BAM-S014 are 3.9% for d_10_, 1.9% for d_50_, and 2.9% for d_90_,(5.2% for d_10_, 2.2% for d_50_, and 8.2% for d_90_ for BAM-D001 with certified values in the same order of magnitude) [25].

**Table 7:** Results of particle size measurements (in µm; d_10_, d_50_, d_90_: percentiles which indicate the size x below which a certain quantity of the sample lies; d_10_ means: 10% of the sample has a particle size below this value)

Line no	d_10_	d_50_	d_90_
**1**	5.81	10.91	18.07
**2**	5.88	11.05	18.60
**3**	6.21	11.16	18.91
**4**	6.28	11.35	19.34
**5**	6.35	11.36	19.40
**6**	6.36	11.42	19.50
**7**	6.39	11.47	19.57
$M$	6.18	11.24	19.06
${s}_{M}$	0.24	0.21	0.56

Although the data for the particle size distribution are of similar quality as in the CRM BAM-D001, the mean values of d_10_, d_50_, and d_90_ are given on the certificate for information only since no homogeneity data for these parameters are available.

### Li isotopic ratio

The lithium isotope ratio (^7^Li/^6^Li) determined in BAM-S014 was characterized using HR-CS-AAS and MC-ICP-MS, ensuring data robustness through complementary measurement techniques. The mean value of the four datasets was 12.34 with a standard deviation of 0.01. Recent studies have shown that HR-CS-AAS, when coupled with suitable data processing strategies, achieves a measurement uncertainty compatible with MC-ICP-MS, typically within ± 1‰ for *δ*^7^Li values [12, 21]. This level of precision is sufficient to detect natural variations in lithium isotope ratios, which commonly span tens of per mil in geological materials and thousands of per mil in laboratory reagents [27]. Such differences can help distinguish lithium from different sources. While the interpretation becomes more complex for material from mixed sources, combining high-precision isotopic data with additional contextual or supply-chain information can support meaningful provenance determinations in practice.

### Value assignment and traceability

The certified mass fractions were calculated as arithmetic averages of the accepted laboratory averages. The respective combined uncertainties were calculated from the spread resulting from the certification inter-laboratory comparison (*u*_ilc_), the uncertainty contributions from possible inhomogeneity (*u*_bb_) of the material, and an estimated uncertainty related to the possible decrease (increase for oxygen) of the respective mass fractions by CO_2_ uptake using Eq. 3.

3 $${u}_{c}= \sqrt{{u}_{\text{ilc}}^{2}+ {u}_{\text{bb}}^{2}+{u}_{\text{stab}}}$$

with

${u}_{ilc}=\sqrt{\frac{{S}_{M}^{2}}{n}}$ uncertainty contribution resulting from inter-laboratory comparison

$n$ number of datasets used for calculating the certified mass fraction of each element

${u}_{stab}$ uncertainty component related to the possible decrease of the respective mass fractions by CO_2_ uptake, assuming that the carbon content increases by a factor of 2 (from 600 to 1200 mg/kg). Assuming that the carbon content doubles (from 0.06 to 0.12%), the weight of the material increases by 0.12%. In this case, the decrease in the mass fraction of Ni, Mn, and Co is approximately 0.032% in absolute terms. An assumed uncertainty contribution to stability of 0.05% absolute for these elements is therefore a very conservative estimate.

The certified mass fractions together with their associated uncertainties are shown in Table 8. The main components of the uncertainty are the contribution of potential inhomogeneity for Li, the contribution of certification interlaboratory comparison uncertainty and potential instability for the major elements Ni, Mn, and Co, and the contribution of certification interlaboratory comparison uncertainty for the other elements.

**Table 8:** Certified mass fractions, uncertainty contributions, and expanded uncertainties (coverage factor *k* = 2) of CRM BAM-S014

	Li	Ni	Mn	Co	O	Al	Cr	Fe	Na	S
	in %					in mg/kg				
***M***_**cert**_	**7.62**	**19.76**	**18.22**	**19.80**	**34.0**	**14.1**	**6.3**	**26**	**512**	**1421**
*u*_ilc_	0.018	0.060	0.065	0.056	0.30	0.806	0.375	1.580	5.49	32.7
*u*_bb_	0.073	0.016	0.012	0.014	0.34	0.468	0.054	0.197	2.38	12.5
*u*_stab_	0.020	0.050	0.050	0.050	0.10	0.040	0.020	0.070	1.40	4.0
***U***	**0.17**	**0.16**	**0.17**	**0.16**	**1.0**	**1.9**	**0.8**	**4**	**13**	**71**

Different calibrants of known purity and specified traceability of their assigned values were used for calibration and all relevant parameters were calibrated. The individual results are therefore traceable to the SI, as it is also confirmed by the agreement among the technically accepted datasets. As the assigned values are combinations of agreeing results individually traceable to the SI, the assigned quantity values themselves are traceable to the SI as well.

Battery manufacturers typically define proprietary acceptance criteria for the elemental composition of Li-NMC cathode materials. These criteria may include maintaining the target stoichiometric ratios among Li, Ni, Mn, and Co to within a narrow relative margin (e.g., ± 0.5–1%) to ensure optimal electrochemical properties, as well as limiting trace impurities (e.g., Fe, Na, Cr) to the mg/kg range to prevent detrimental effects on battery cycling performance and safety [1, 3, 8]. Although such specific acceptance thresholds are not publicly disclosed and can differ among producers, the underlying principle remains consistent: Reducing compositional variability and maintaining a stringent purity profile are crucial for achieving stable capacity, long cycle life, and overall product reliability.

The results of the round-robin test demonstrated that the CRM is suitable for the accurate and reliable determination of elemental composition in Li-NMC cathode materials. The CRM showed excellent homogeneity and traceability, and the analytical results obtained from different laboratories were consistent and comparable. The CRM BAM-S014 allows laboratories to benchmark their analytical methods. Laboratories can thus confirm that their measurement capabilities align with the precision and accuracy required by manufacturer’s criteria. In other words, while the CRM does not establish the acceptance limits, it supports laboratories and manufacturers in confidently verifying that their materials meet the exacting standards demanded by the rapidly evolving lithium-ion battery market.

## Conclusion and outlook

The CRM BAM-S014 (NMC 111) is the first certified reference material for Li-based cathode materials. In addition to NMC 111, materials with a lower cobalt content such as NMC 622 or NMC 811 are increasingly being used as well as Li-based materials with another stoichiometry such as lithium iron phosphate. Certified reference materials are also required for these materials. An NMC 811 CRM is currently under development at our institute.

As electromobility continues to grow, the production of batteries will increase, and more used lithium-ion batteries will be generated/accumulated. Recycling cycles have to be developed in order to recover as many of the materials and elements used in the batteries as possible. Analytical methods and reference materials will be required to monitor the quality of the recycled material and ensure the accurate determination of the value-determining elements in particular. In the long term, it is necessary to investigate the complete life cycle of battery materials.

Analyzing black mass, the concentrated material containing valuable metals like lithium, cobalt, nickel, manganese, aluminum, and copper extracted from used lithium-ion batteries during the recycling process requires the development of specialized digestion and determination methods to quantify these critical elements accurately [28]. In order to ensure the quality of the analysis results here too, the development of reference materials based on black mass is planned, and initial analytical tests have already taken place.
